# Efficacy and safety of combined stent retriever and contact aspiration vs. stent retriever alone on revascularization in patients with acute ischemic stroke: a systematic review and meta-analysis

**DOI:** 10.3389/fneur.2024.1365876

**Published:** 2024-06-04

**Authors:** Wei Li, Guo-hui Lin, Hong-hong Li, Peng-bo Zhou, Yue-yang Chen, Hong-tao Sun, He-cheng Chen

**Affiliations:** ^1^The First School of Clinical Medical, Lanzhou University, Lanzhou, China; ^2^Tianjin Key Laboratory of Neurotrauma Repair, Characteristic Medical Center of People’s Armed Police Forces, Tianjin, China; ^3^Gansu Provincial Maternity and Chlid-Care Hospital, Lanzhou, China; ^4^Department of Cerebrovascular Disease, Gansu Provincial People’s Hospital, Lanzhou, China

**Keywords:** combined stent retriever and contact aspiration, stent retriever alone, acute occlusion of large vessels, acute ischaemic stroke, meta-analysis

## Abstract

**Objective:**

Whether the efficacy of combined stent retriever and contact aspiration (S + A) is superior to stent retriever (S) alone for revascularisation in patients with large vessel occlusive stroke remains uncertain. The aim of this meta-analysis was to assess the safety and efficacy of combined stent retriever and contact aspiration for the treatment of acute ischaemic stroke with large vessel occlusion by comparing it with stent retriever alone.

**Methods:**

We systematically searched the PubMed, Embase, Web of Science, and The Cochrane Library databases for randomised controlled trials and observational studies (case-control and cohort studies) published before 1 October 2023 comparing the efficacy of combined stent retriever and contact aspiration versus tent retriever alone in patients with large vessel occlusive stroke. The end point of the primary efficacy observed in this meta-analysis study was the rate of first pass nearly complete or complete recanalisation (mTICI 2c-3). Secondary effectiveness nodes were: rate of first pass successful recanalisation (mTICI 2b-3), rate of near-complete or complete recanalisation of the postoperative vessel, rate of successful recanalisation of the postoperative vessel, and MRS 0–2 within 90 days. Safety endpoints were interoperative embolism, symptomatic intracranial haemorrhage, and mortality within 90 days.

**Results:**

A total of 16 studies were included in the literature for this meta-analysis, with a total of 7,320 patients (S + C group: 3,406, S group: 3,914). A comprehensive analysis of the included literature showed that combined stent retriever and contact aspiration had a higher rate of near-complete or complete recanalisation of the postoperative vessel [OR = 1.53, 95% CI (1.24, 1.88), *p* < 0.0001] and rate of successful recanalisation of the postoperative vessel compared to stent retriever alone [OR = 1.83, 95% CI (1.55, 2.17), *p* < 0.00001]; there were no statistically significant differences between the two groups in terms of the rate of first pass nearly complete or complete recanalisation [OR = 1.00, 95% CI (0.83, 1.19), *p* = 0.96], rate of first pass successful recanalisation [OR = 1.02, 95% CI (0.85, 1.24), *p* = 0.81], interoperative embolism [OR = 0.93, 95% CI (0.72, 1.20), *p* = 0.56], symptomatic intracranial haemorrhage [OR = 1.14, 95% CI (0.87, 1.48), *p* = 0.33], MRS 0–2 within 90 days [OR = 0.89, 95% CI (0.76, 1.04), *p* = 0.14] and mortality within 90 days [OR = 1.11, 95% CI (0.94, 1.31), *p* = 0.22].

**Conclusion:**

Combined stent retriever and contact aspiration has a higher rate of postprocedural revascularisation (mTICI 2c-3/mTICI 2b-3) compared with stent retriever alone in patients with large vessel occlusion stroke. In addition, it was not superior to stenting alone in terms of the rate of first pass recanalisation (mTICI 2c-3/mTICI 2b-3), interoperative embolisation, symptomatic intracranial haemorrhage, good functional prognosis within 90 days and mortality within 90 days.

## Introduction

Stroke is a serious threat to human health and is the leading cause of disability and death in adults, with ischaemic stroke accounting for approximately 87% of stroke incidence (1). The treatment of ischemic stroke mainly lies in early opening of occluded blood vessels, restoring blood flow, and maximally saving the ischemic penumbra. Especially for patients with large-vessel occlusive stroke, vascular opening and reconstruction of blood flow are crucial to the patient’s prognosis.

Endovascular mechanical thrombolysis is now regarded as the standard of care for stroke patients with large vessel occlusion, and its safety and efficacy have been confirmed by five randomised controlled trials (2). Currently, endovascular mechanical embolisation is mainly performed by stenting alone and contact aspiration, with the ideal goal of completely opening the occluded vessel in a short period of time, improving clinical prognosis and reducing complications. Some clinical trials have found successful recanalisation rates of only 58–88% with stenting and two randomised controlled trials have shown similar angiographic and clinical outcomes between stenting and contact aspiration (3–5). With the widespread use of both methods of embolisation, technical limitations have been identified, such as thrombus rupture and escape during stent and suction catheter retrieval, leading to embolisation of distal vessels, incomplete or failed revascularisation, and vessel rupture during stenting leading to higher levels of intracranial haemorrhage. Therefore, the innovation of endovascular mechanical thrombolysis techniques is one of the main ways to increase successful revascularisation after thrombolysis. Both European and American scholars have also recommended clinical trials to determine the optimal strategy for the use of mechanical retrieval devices to achieve the highest reperfusion success rates (6, 7).

The combined stent retriever and contact aspiration has been reported several times in recent years. Several studies have found that stenting combined with thrombus aspiration is more effective in successful revascularisation (8–11). However, a randomised controlled trial by Lapergue et al. (12) demonstrated that in patients with acute ischaemic stroke due to large vessel occlusion, combined stent retriever and contact aspiration did not significantly improve the rate of near-total or total recanalisation at the end of endovascular treatment procedures compared with stenting alone (eTICI 2c/3). In a comparative study by Huo et al. (13) that included a Chinese population, it was also found that SR + CA treatment was not superior to SR alone in terms of final revascularisation level, first revascularisation level and good prognosis for 90-day clinical outcome. The results remain uncertain as to whether the efficacy and safety of combined stent retriever and contact aspiration is superior to stent retriever alone. The aim of this study was to assess the safety and efficacy of combined stent retriever and contact aspiration by performing a meta-analysis of randomised controlled trials and observational studies comparing the efficacy of combined stent retriever and contact aspiration versus stent retriever alone in large-vessel occlusive stroke.

## Methods

### Search strategy

This meta-analysis was performed according to the PRISMA guidelines. We systematically searched PubMed, Embase, Web of Science, and The Cochrane Library databases for randomised controlled trials and observational studies (case-control studies and cohort studies) published before 1 October 2023 comparing the efficacy of combined stent retriever and contact aspiration versus stent retriever alone in patients with acute ischaemic stroke. A literature search was conducted independently by 2 researchers and we used a combination of the following terms: ischemic strokes (mesh), ischaemic stroke, cryptogenic ischemic stroke, acute large vessel occlusion, embolism stroke, cryptogenic, wake up stroke, acute ischemic stroke, aspiration thrombectomy, thrombectomies, aspiration, thrombectomies, percutaneous aspiration, contact aspiration, stent retriever, stent retriever alone. References generated from these searches were imported into the reference manager EndNote X9.3.1 (Thompson Reuters, Philadelphia, PA) and duplicate references were removed. Then, journal article titles and abstracts were systematically screened independently by 2 researchers according to inclusion and exclusion criteria.

### Inclusion criteria

(1) Patients with confirmed acute ischaemic stroke or acute large vessel occlusive stroke; (2) endovascular treatment: combined stent retriever and contact aspiration, stent thrombolysis alone; (3) comparative data on the efficacy of the two treatment groups can be provided explicitly in the literature: combined stent retriever and contact aspiration group and stent retriever alone group. (4) Randomised controlled trials and observational studies (case-control studies and cohort studies).

### Exclusion criteria

(1) Conference abstracts, letters, reviews, correspondence, animal experiments and unpublished studies; (2) studies with duplicate or overlapping data; (3) lack of studies with follow-up data beyond hospitalisation; (4) literature that was unable to provide comparative data on the efficacy of the two treatment groups: combined stent retriever and contact aspiration group and stent retriever alone group; (5) sample sizes were all case series of <10 patients.

### Data extraction and efficacy indicators

Data for each eligible literature were extracted independently by 2 researchers, and any disagreements were resolved through discussion and consultation with a 3rd senior neurosurgeon. Basic information such as first author’s name + year of publication, study design, sample size, mean age, sex ratio, site of occlusion, and endovascular treatment modality were extracted using a predefined form. The end point of the primary efficacy observed in this meta-analysis study was the rate of first pass nearly complete or complete recanalisation (mTICI 2c-3). Secondary effectiveness nodes were: rate of first pass successful recanalisation (mTICI 2b-3), rate of near-complete or complete recanalisation of the postoperative vessel, rate of successful recanalisation of the postoperative vessel, and MRS 0–2 within 90 days. Safety endpoints were interoperative embolism, symptomatic intracranial haemorrhage, and mortality within 90 days.

### Literature quality assessment

Each of the 2 trained researchers read all literature titles and abstracts, first screening out literature that clearly did not meet the inclusion criteria, and then reading the full text of the literature further to initially identify literature that could be included in the study. Finally, the two researchers’ screening results were cross-checked, and the two evaluators discussed the questionable literature and combined third-party opinions to decide whether to include it or not. The quality of randomised controlled trials was evaluated using the Cochrane Risk of Bias tool, and the quality of observational studies was evaluated using the Newcastle–Ottawa Scale.

### Statistical analysis

Statistical analyses were performed using Review Manager (v.5.3), and differences were considered statistically significant at *p* ≤ 0.05 if not explicitly stated. We calculated the odds ratio (OR) of categorical variables using a random-effects model, and heterogeneity was evaluated using chi-square tests and *I*^2^ tests, and we considered data to be significantly heterogeneous when *I*^2^ > 50%, and we performed meta-analysis using a random-effects model, otherwise, a fixed-effects model was performed. Sensitivity analyses were performed by omitting studies one by one to assess the effect of each study on the overall outcome. Symmetry was assessed using Begg’s and Egger’s tests, and significant publication bias was defined as *p* < 0.1, and publication bias was assessed with sensitivity analysis using STATA (v.12).

## Results

### Search results and selection of research subjects

**Figure 1:**
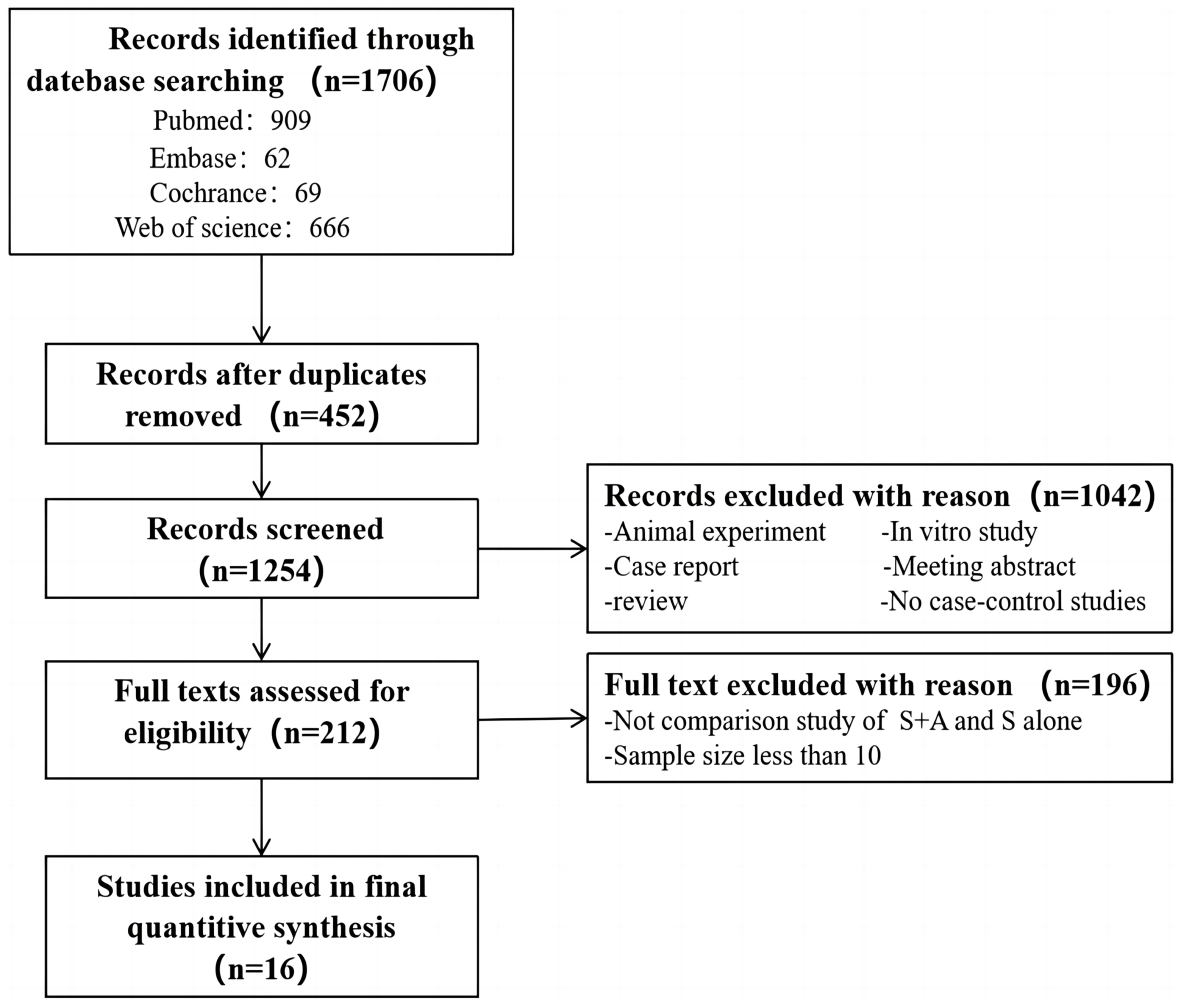
Flow chart of the search and inclusion of literature.

A search of databases was conducted to identify 1,076 documents (Pubmed: 909, Embase: 62, Cochrance: 69, Web of Science: 666), of which 452 duplicates were excluded. An additional 1,042 papers were excluded by screening the titles and abstracts of the shortlisted papers, and the remaining 212 papers were read in full and in detail to determine whether they met the inclusion/exclusion criteria. Eventually, 16 eligible papers were included in this meta-analysis, as shown in Figure 1.

### Basic characteristics of the research object

A total of 7,320 patients from 16 (12–27) studies (1 randomised controlled trial and 15 observational studies) were enrolled in this study, including 3,406 with combined stent retriever and contact aspiration and 3,914 with stenting alone. The characteristics of the demographics regarding the type of literature included in the study are shown in Table 1.

**Table 1:** Basic characteristics of the included studies.

Study	Design	Sample size		Mean age, years (S + C/S)	Gender (M/F)		Occlusion site	Endovascular therapy
		S + C	S		S + C	S		
Hesse et al. (14)	Observation study	184	102	75/74	103/81	57/45	Anterior circulation	S/S + A/BGC
Procházka et al. (15)	Observation study	64	196	69/69	34/30	90/106	LVO	S/Solumbra
Colby et al. (16)	Observation study	106	215	72.5/71	52/54	104/111	ICA, MCA, ACA	S/S + A/Solumbra
Kim et al. (17)	Observation study	42	49	71.3/69.0	21/21	22/27	ICA bifurcation, M1, M2	S/T + A/BGC
Di Maria et al. (18)	Observation study	339	550	NA	NA		M1-MCA, intracranial ICA	S/S + A/BGC
Lapergue et al. (12)	RCT	203	202	73.6/73.3	99/104	86/116	Intracranial ICA, M1, M2	S/S + A/BGC
Blasco et al. (19)	Observation study	128	273	73.5/79	54/74	133/140	Carotid terminus, MCA-M1	S/S + A/BGC
Maïer et al. (20)	Observation study	1,111	406	72.8/69.2	531/580	200/206	Intracranial ICA, M1	S/S + A
Mohammaden et al. (21)	Observation study	148	148	65.8/66.6	82/66	66/82	ICA, MCA-M1, MCA-M2	Trevo/Solitaire/Embotrap
Okuda et al. (22)	Observation study	240	128	81/77	NA		ICA, MCA-M1, MCA-M2	S/S + A/BGC
Perez-Garcia et al. (23)	Observation study	67	67	75.6/74.2	41/26	40/27	MCA-M2	S/S + A
Abdelrady et al. (24)	Observation study	35	35	68.7/65.89	25/10	20/15	Basilar artery occlusion	S/S + A
Abecassis et al. (25)	Observation study	230	180	NA	NA		Anterior /posterior circulation	S/S + A
Bala et al. (26)	Observation study	223	165	71/68	105/118	81/84	ICA, MCA-M1, MCA-M2	S/S + A
Huo et al. (13)	Observation study	164	1,069	69/65	97/67	684/385	ICA, M1, M2	S/S + A
Xu et al. (27)	Observation study	122	129	62/64	70/52	70/59	Observation study	S/S + A

S, stent retrieval; S + C, stent retrieval + contact aspiration; ICA, internal carotid artery; M1, middle cerebral artery-1; M2, middle cerebral artery-2; T, Trevo; NA, not available; BGC, balloon-guide catheter.

### Quality assessment of included literature

A total of 16 studies were included (12–27), 1 study was an RCT, evaluating the quality of randomised controlled trials using the Cochrane Risk of Bias tool, and 15 studies were observational, evaluating the quality of non-randomised controlled trials using the NOS quality assessment. In conclusion, the quality scores of the included literature were high, describing the selection of the study population and the comparability between groups.

### Efficacy and safety

#### The rate of first pass near complete or complete recanalisation (mTICI 2c-3)

**Figure 2:**
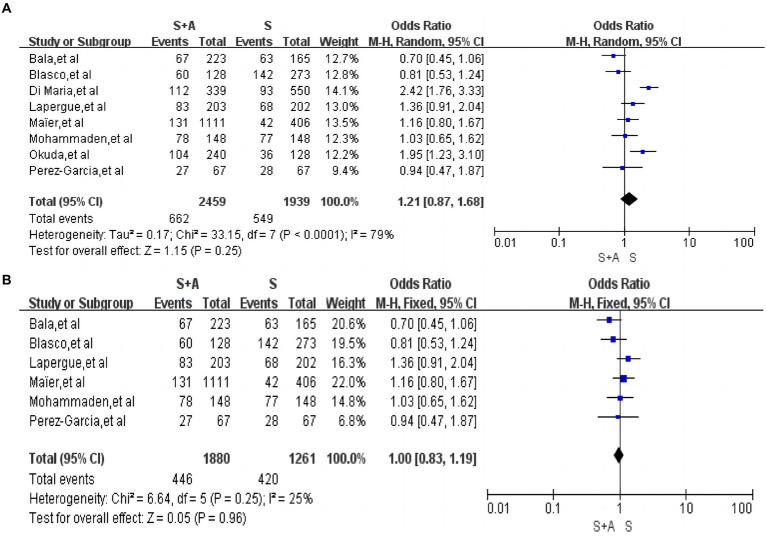
Forest plot and meta-analysis of the rate of first pass near complete or complete recanalisation.

In the evaluation of first pass near-complete or complete recanalization rates, a total of eight (12, 18–23, 26) studies were included with high heterogeneity (*I*^2^ = 79%, *p* < 0.0001). The combined stent retriever and contact aspiration (S + A) totaled 2,459, with 662 (26.9%, 662/2,459) patients with first pass near-complete or complete recanalization. The stent retriever group (S) totaled 1,939, with 549 (28.3%, 549/1,939) patients with first pass near-complete or complete recanalization. Adoption of random effects model. There was no statistically significant difference between the S + A group and the S group in terms of first pass nearly complete or complete recanalization rate [OR = 1.21, 95% CI (0.87, 1.68), *p* = 0.25], as shown in Figure 2A. After the exclusion of two studies by Di Maria et al. (18) and Okuda et al. (22) the heterogeneity between the included literature was significantly lower (*I*^2^ = 25%, *p* < 0.25). The rate of first pass near-complete or complete recanalization was 23.7% (446/1,880) in the S + A group and 33.3% (420/1,261) in the S group, which did not affect the final outcome [OR = 1.00, 95% CI (0.83, 1.19), *p* = 0.96], as shown in Figure 2B.

#### The rate of first pass successful recanalisation (mTICI 2b-3)

**Figure 3:**
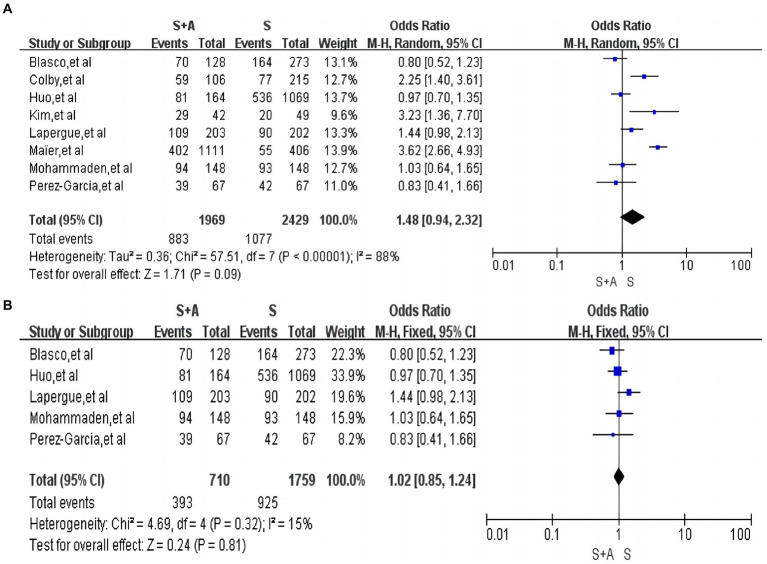
Forest plot and meta-analysis of the rate of first pass successful recanalisation.

In terms of the rate of first pass successful recanalisation, a total of eight articles were included (12, 13, 16, 17, 19–21, 23), with high heterogeneity (*I*^2^ = 88%, *p* < 0.0001). The rate of first pass successful revascularization was 44.8% (883/1,969) in the S + A group and 44.3% (1,077/2,429) in the S group. There was no statistically significant difference between the S + A group and the S group in terms of the rate of first pass successful revascularization [OR = 1.48, 95% CI (0.94, 2.32), *p* = 0.09], as shown in Figure 3A. Heterogeneity was reduced after the exclusion of three studies by Colby et al. (16), Kim et al. (17), and Maïer et al. (20) (*I*^2^ = 15%, *p* = 0.32). The rate of first pass successful recanalization was 55.3% (393/710) in the S + A group and 52.6% (925/1,759) in the S group, and there was no statistically significant difference between the two groups [OR = 1.02, 95% CI (0.85, 1.24), *p* = 0.81], as shown in Figure 3B.

#### The rate of near-complete or complete recanalization after operation

**Figure 4:**
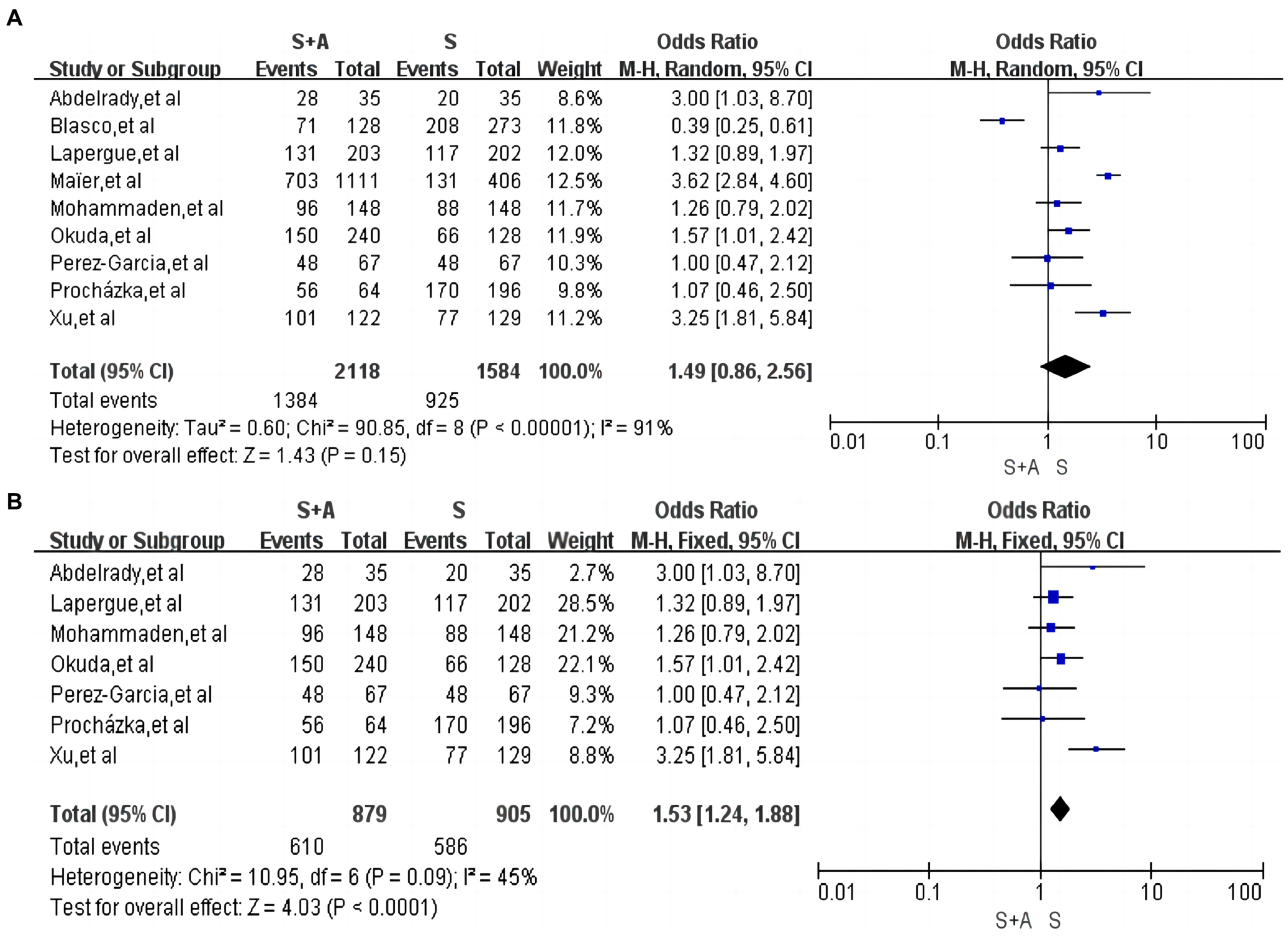
Forest plot and meta-analysis of the rate of near-complete or complete recanalization after operation.

In terms of the rate of near-complete recanalization or complete recanalization after the operation, a total of nine articles were included (12, 15, 19–24, 27), with high heterogeneity (*I*^2^ = 91%, *p* = 0.00001). A total of 2,118 persons were included in the S + A group, and 1,384 (65.3%, 1,384/2,118) were near-complete recanalization or complete recanalization of the vessels after the operation; a total of 1,584 persons were included in group S. Near-complete recanalization or complete recanalization of vessels after operation was achieved in 925 individuals (58.4%, 925/1,584). A random-effects model was used. There was no statistically significant difference between the S + A group and S group in terms of the rate of near-complete recanalization or complete recanalization of the vessels after the operation [OR = 1.49, 95% CI (0. 86, 2.56), *p* = 0.15], as shown in Figure 4A. However, after excluding one study by Maïer et al. (20) the remaining studies were analyzed together with low heterogeneity (*I*^2^ = 45%, *p* = 0.09). The rate of near-complete recanalization or complete recanalization after the operation was 69.4% (610/879) in the S + A group, and the rate of near-complete recanalization or complete recanalization after the operation was 64.8% (586/905) in the S group. The S + A group was superior to the S group [OR = 1.53, 95% CI (1.24, 1.88), *p* < 0.0001], as shown in Figure 4B.

#### The rate of successful recanalization after operation

**Figure 5:**
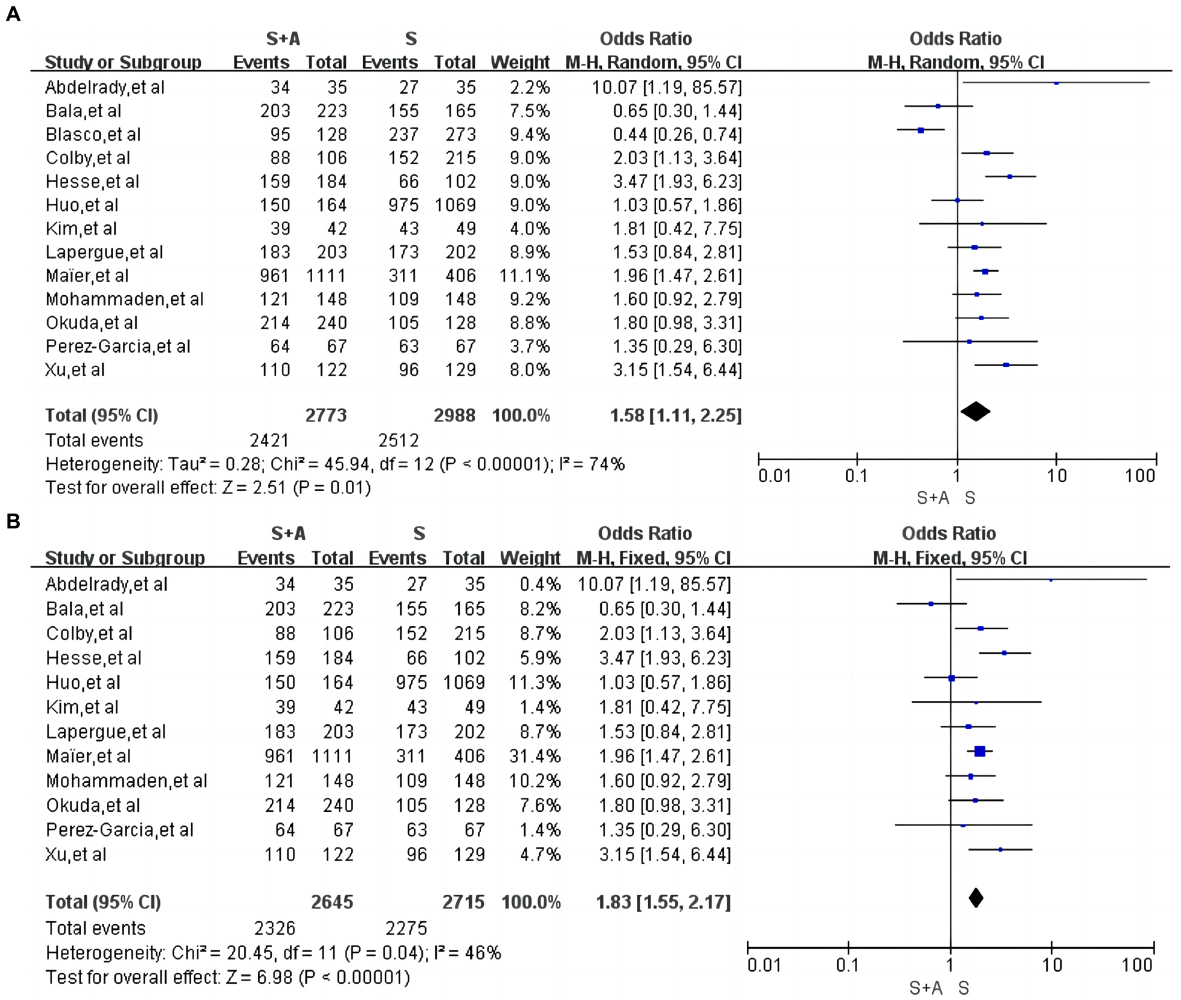
Forest plot and meta-analysis of the rate of successful recanalization after operation.

In terms of the rate of successful recanalization after operation, a total of 13 articles were included (12–14, 16, 17, 19–24, 26, 27), with high heterogeneity (*I*^2^ = 74%, *p* = 0.0001). Use of random effects models. A total of 2,773 persons were included in the S + A group, with 2,421 successful recanalization after operation (87.3%, 2,421/2,773); a total of 2,988 persons were included in the S group, with 2,512 successful recanalization after operation (84.1%, 2,512/2,988). In terms of the rate of successful revascularization after operation, the S + A group was superior to the S group [OR = 1.58, 95% CI (1.11, 2.25), *p* = 0.01], as shown in Figure 5A. Heterogeneity was reduced after the exclusion of one study by Blasco et al. (19) (*I*^2^ = 46%, *p* = 0.04). The rate of successful recanalisation after the operation was 87.9% (2,326/2,645) in the S + A group and 83.8% (2,275/2,715) in the S group, with a statistically significant difference between the two groups [OR = 1.83, 95% CI (1.55, 2.17), *p* < 0.00001], as shown in Figure 5B.

#### MRS 0–2 within 90 days

**Figure 6:**
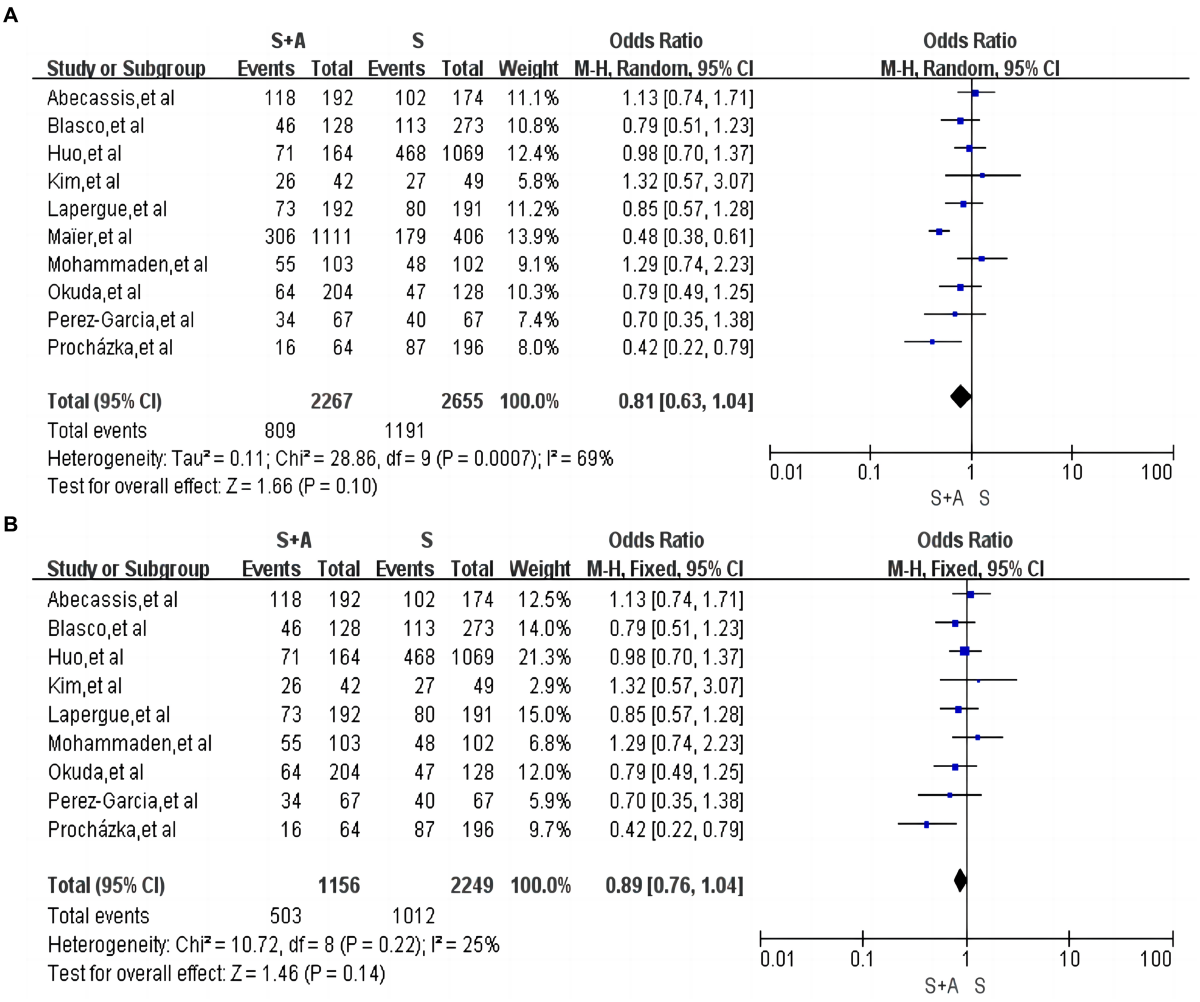
Forest plot and meta-analysis of MRS 0–2 within 90 days.

In terms of 90-day good functional prognosis, a total of 10 papers were included (12, 13, 15, 17, 19–23, 25) with high heterogeneity (*I*^2^ = 69%, *p* = 0.0007). Use of random effects models. The 90-day good functional prognosis was 35.7% (809/2,267) in the S + A group and 44.9% (1,191/2,655) in the S group, and the difference between the two groups was not statistically significant [OR = 0.81, 95% CI (0.63, 1.04), *p* = 0.1], as shown in Figure 6A. Heterogeneity was significantly lower after excluding one study by Maïer et al. (20) (*I*^2^ = 25%, *p* = 0.22). The 90-day good functional prognosis was 43.5% (503/1,156) in the S + A group and 44.9% (1,012/2,249) in the S group, and the difference between the two groups was still not statistically significant [OR = 0.89, 95% CI (0.76, 1.04), *p* = 0.14], as shown in Figure 6B.

#### Interoperative embolism

**Figure 7:**
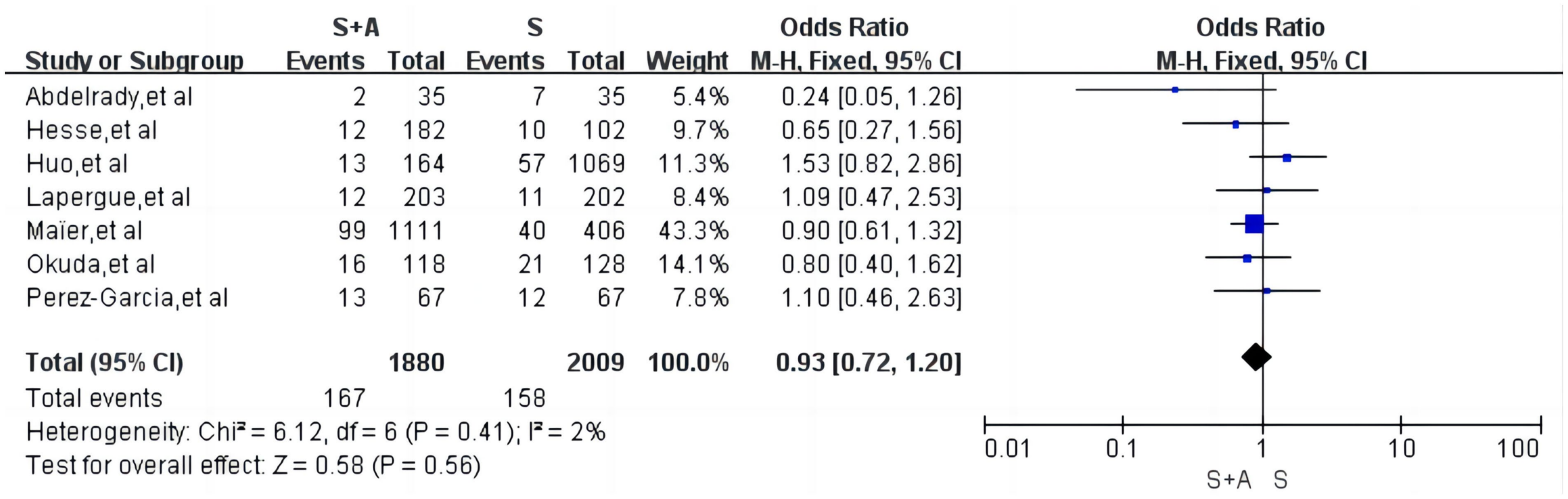
Forest plot and meta-analysis of interoperative embolism.

With regard to interoperative embolism, a total of 7 articles were included (12–14, 20, 22–24), low heterogeneity (*I*^2^ = 2%, *p* = 0.41), with an interoperative embolism rate of 8.9% (167/1,880) in the S + A group and 7.9% (158/2,009) in the S group, and the difference between the two groups was not statistically significant [OR = 0.93, 95% CI (0.72, 1.20), *p* = 0.56], as shown in Figure 7.

#### Symptomatic intracranial haemorrhages

**Figure 8:**
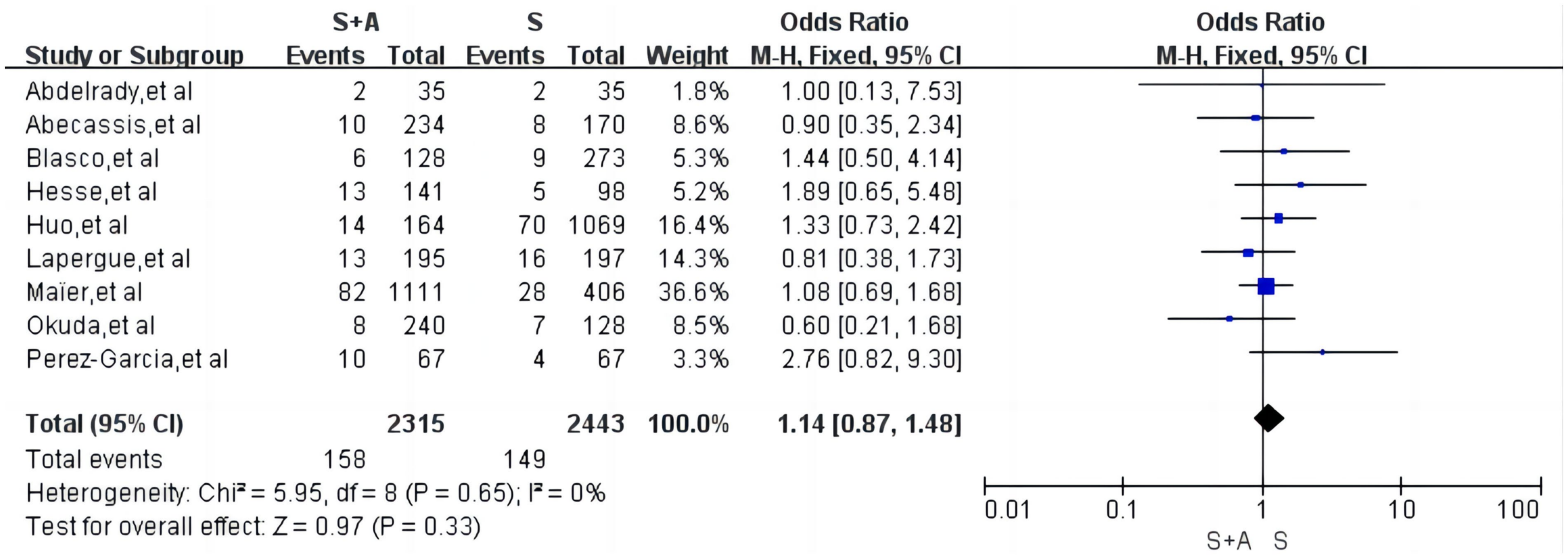
Forest plot and meta-analysis of symptomatic intracranial haemorrhages.

Regarding symptomatic intracranial haemorrhage, a total of 9 articles were included (12–14, 19, 20, 22–25), with low heterogeneity (*I*^2^ = 0%, *p* = 0.65), and the rate of symptomatic intracranial haemorrhage in the S + A group was 6. 8% (158/2,315) and the rate of symptomatic intracranial haemorrhage in the S group was 6.1% (149/2,443), and the difference between the two groups was not statistically significant [OR = 1.14, the 95% CI (0.87, 1.48), *p* = 0.33], as shown in Figure 8.

#### Mortality within 90 days

**Figure 9:**
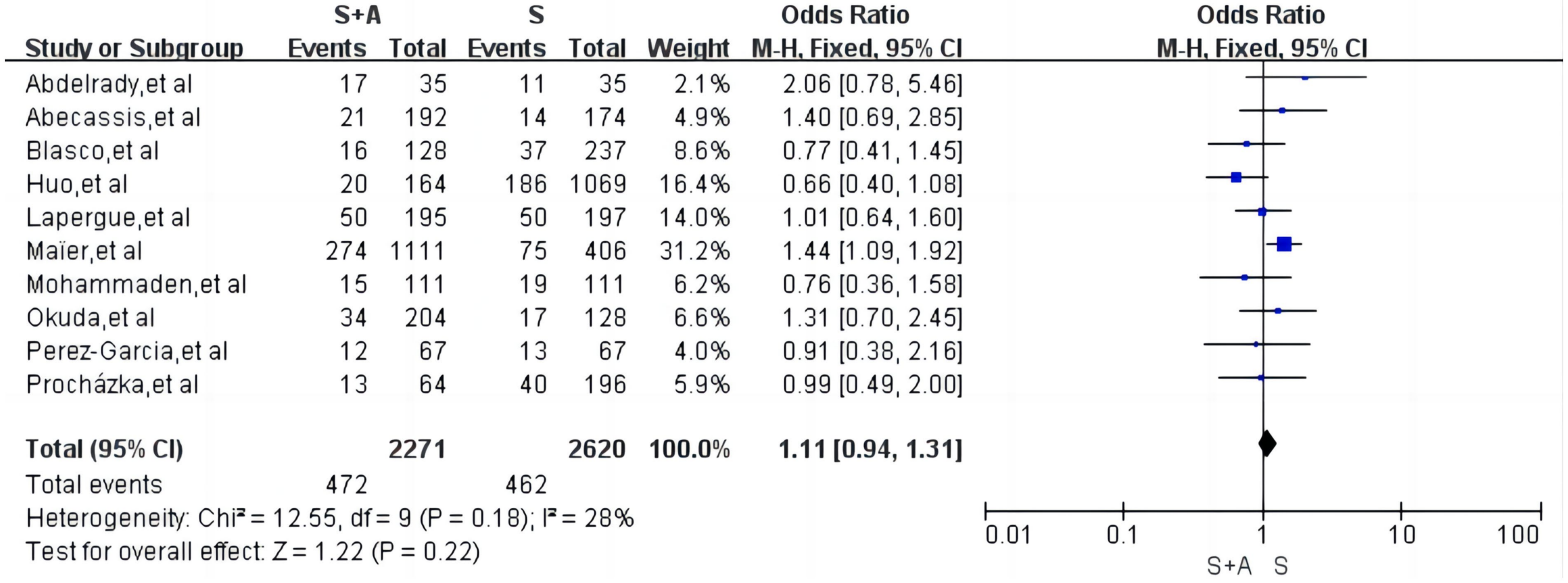
Forest plot and meta-analysis of mortality within 90 days.

Regarding mortality within 90 days, a total of 10 articles were included (12, 13, 15, 19–25) with low heterogeneity (*I*^2^ = 28%, *p* = 0.18). The mortality rate within 90 days was 20.8% (472/2,271) in group S + A and 17.6% (462/2,620) in group S. The difference between the two groups was not statistically significant [OR = 1.11, 95% CI (0.94, 1.31), *p* = 0.22], as shown in Figure 9.

#### Subgroup analysis based on different embolization sites

**Figure 10:**
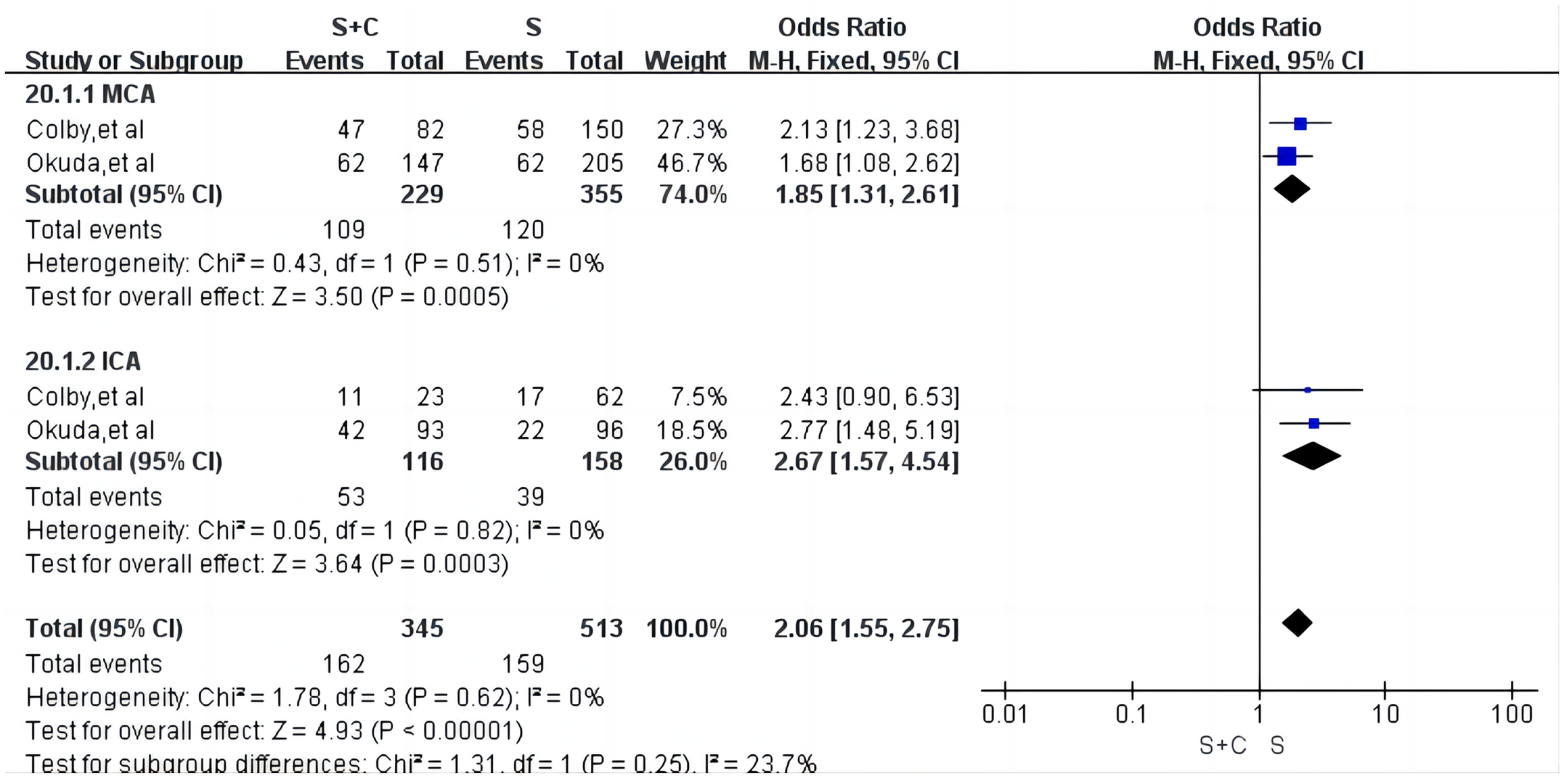
Forest plot and meta-analysis of subgroup analysis based on different sites of embolism.

We conducted a subgroup analysis on the first successful vascular recanalization at different embolization sites. For the middle cerebral artery occlusion segments, a total of two articles were included (16, 22), with low heterogeneity (*I*^2^ = 0%, *p* = 0.51). The first successful vascular recanalization rate was 47.6% in the S + A group compared to 33.8% in the S group, with the S + A group significantly outperforming the S group [OR = 1.85, 95% CI (1.31, 2.61), *p* = 0.0005, as shown in Figure 10]. In the internal carotid artery occlusion segments, there was low heterogeneity (*I*^2^ = 0%, *p* = 0.62). The first successful vascular recanalization rate was 45.7% in the S + A group versus 24.7% in the S group, with the S + A group significantly outperforming the S group [OR = 2.67, 95% CI (1.57, 4.54), *p* = 0.0003, as shown in Figure 10].

#### Sensitivity analyses and publication bias

In this meta-analysis, the results of sensitivity analyses comparing the efficacy of combined stent retriever and contact aspiration versus stent retriever alone were consistent with the results of the combined analyses; we used Begg’s method and Egger’s method test to assess the effect of publication bias, and the funnel plots were both symmetric and there was no clear evidence of publication bias.

## Discussion

Combined stent retriever and contact aspiration has been widely reported in recent years with the aim of increasing the successful recanalisation rate in patients with large vessel occlusion, reducing interoperative complications such as embolism and bleeding, and improving the functional prognosis of patients by combining stenting and contact aspiration, but the efficacy and feasibility of combined stent retriever and contact aspiration is still controversial. The use of combined techniques may increase the cost of patient care, and Meder et al. (28) found that switching from stenting alone to stenting combined with aspiration may increase the cost of mechanical extraction of boluses by approximately 30% at their institution. Moreover, studies have already reported (12, 13) that stenting combined with aspiration did not increase the rate of successful post-procedural recanalisation, the rate of first-time recanalisation, and did not improve the functional prognosis within 90 days in patients with large vessel occlusion compared to stenting alone. Therefore, it is necessary to analyse the combined stent retriever and contact aspiration in comparison with stent retriever alone. In total, this Meta-analysis included 16 papers of comparative studies of the two treatment methods involving 7,320 patients, which were synthesised to show that: in terms of the major effectiveness nodes (mTICI ≥2C), combined stent retriever and contact aspiration did not demonstrate a significant advantage over stent retriever alone. However, in the secondary effectiveness nodes, combined stent retriever and contact aspiration was superior to stent retriever alone in terms of near-complete or complete recanalisation of the vessel post-procedure (mTICI ≥2C), and successful recanalisation of the vessel post-procedure (mTICI ≥2b). However, it did not show a significant advantage in terms of first pass successful recanalisation (mTICI ≥2b) and good functional prognosis at 90 days. With regard to safety, no significant differences were seen in interoperative embolism, symptomatic intracranial haemorrhage, and mortality within 90 days with combined stent retriever and contact aspiration compared with stent retriever alone.

Mechanical thrombectomy has significant efficacy in patients with acute large-vessel occlusive ischaemic stroke (6), but the efficacy of the two popular techniques of mechanical thrombectomy has not fully achieved the expected goals of the treatment, and has not maximised the benefits for stroke patients. Therefore, innovations in mechanical embolisation techniques are a constant pursuit for neurointerventionalists. It has been reported in the literature (11, 29–32) that the combined technique improves the rate of first pass successful and post-procedural recanalisation compared to a single device, and its main technical advantage lies in the fact that in the combined technique, the stent retriever, located distal to the clot, and the aspiration catheter, located proximal to the clot, allow us to capture clots from both sides, and the large-bore aspiration catheter also allows for the direct removal of additional thrombus, and in addition, the interoperative catheter is continuously negative pressure to capture the proximal thrombus mass, thus reducing the incidence of thrombus fragmentation during stent withdrawal. However, the results of this meta-analysis study found no significant differences between combined embolisation compared with stenting alone in terms of mTICI grade ≥2c and mTICI grade ≥2b after first pass recanalisation. However, in terms of postoperative recanalisation mTICI grade ≥2c and mTICI grade ≥2b, combined thrombolysis improved the rate of successful recanalisation. We speculate that the main reasons for the increased rates of complete and successful recanalisation after operation may be the following: Firstly, technique crossover, which is more prevalent in combined embolisation, with the incidence of technique crossover being as high as 30–45% in retrospective study series (19, 22). Secondly, thrombus composition and size, for large and hard thrombi, the two different techniques may not show a significant difference after one operation, but as the number of MTs increases, the advantages of the combined technique appear. Thirdly, thrombus sites differed, in terms of occlusion sites, the incidence of successful recanalisation was significantly higher with the combined technique than with the alone technique in ICA and M2 occlusions, which may be mainly attributed to the large, hard clots commonly seen in ICA (33), and the smaller vessel diameter of the M2 which reduces dead space with the aspiration catheter, thus increasing the aspirational force (34).

This meta-analysis found no difference between the two groups in terms of overall first successful vascular recanalization and post-operative successful vascular recanalization. However, subgroup analysis based on different embolization sites revealed that for both MCA and ICA segments, the rate of initial successful vascular recanalization using combined techniques was significantly higher compared to the standalone stent retriever technique, with statistical differences being more pronounced in the ICA segment. However, these results could be biased due to the small number of studies included. Schartz et al. (35) also found that the combined thrombectomy group had a higher rate of first successful recanalization compared to the standalone stent retriever group, but there were no significant differences in the rate of final successful recanalization. The recent ASTER 2 clinical randomised controlled trial similarly reviewed the efficacy of the combined technique versus stenting alone and also found no difference between the two groups in terms of first-pass recanalisation (mTICI ≥2b, mTICI ≥2b) (12). Huo et al. (13) also showed no difference between the combined technique and stenting alone in terms of first pass recanalisation in a study conducted in a Chinese population. However, in the ASTER 2 clinical randomised controlled trial and the Huo et al. (13) study, the combined embolisation technique did not show an advantage in terms of postoperative recanalisation.

The first pass effect (mTICI ≥2b) is considered to be strongly associated with a favourable prognosis in patients after mechanical embolisation, mainly due to the fact that fewer passes lead to fewer complications and better outcomes are achieved if complete reperfusion is achieved after the first pass (36). This meta-analysis showed no difference between the two groups in terms of 90-day good functional prognosis (MRS ≤2), which is consistent with the results of several previous studies (12, 13, 22, 23, 35). Moreover, the present Meta-analysis demonstrated that there was no difference in the first pass effect between the two groups. In terms of safety, this meta-analysis focused on a pooled analysis of interoperative embolism, symptomatic intracranial haemorrhage, and mortality at 90 days, and we found that the combined technique did not increase the number of procedural complications or mortality at 90 days compared with stenting alone. Although, this result is consistent with the results of some previous studies (12, 13, 22), Hesse et al. (14) found that the combined technique group had a lower rate of interoperative embolism. a study by Xu et al. (27) also showed that the combined technique group had a lower rate of disease-related adverse events (including all-cause mortality). So I had to go ahead and consider the use of the two-queue balloon-guide catheter (BGC) in the original article that was included. The use of BGC reduces the difference in efficacy between the combined technique and stenting alone, as BGC stops blood flow and reduces thrombus fragmentation and distal embolisation (37). Kurre et al. (38) reported a reduction in distal embolisation rates (14.6 to 3.3%) by the addition of an intermediate aspiration catheter in the SR without the use of a BGC. Bourcier et al. (39) showed that BGC did not lead to better reperfusion and clinical outcomes when stenting combined with aspiration compared to no BGC, and that the use of BGC may have diminished the role of the intermediate aspiration catheter.

Some limitations should be highlighted when interpreting the results. First, most of the included studies were retrospective with limited follow-up, which may overestimate the effect size of the results and limit the interpretation of the pooled data. Second, even after adjusting for differences between groups at baseline, the possibility of confounding by measured or unmeasured variables cannot be ruled out. Third, in the absence of blinded assessments, the assessment of clinical outcomes may be biased. In addition, the included studies used a variety of devices (e.g., BGC, guide catheters, aspiration catheters, different stent retrievers), which contributed to the heterogeneity.

## Conclusion

Combined stent retriever and contact aspiration did not increase the rate of first pass near-complete or complete recanalisation and first pass successful recanalisation in patients with large-vessel occlusion stroke compared with stent retriever alone, but it did increase the rate of postprocedural near-complete or complete recanalisation and postprocedural successful recanalisation. However, no significant advantage was seen in terms of good functional prognosis at 90 days, interoperative embolism, symptomatic intracranial haemorrhage, or mortality at 90 days. This result still needs to be further confirmed by additional large-sample, multicenter, prospective randomized controlled trials.

## Data availability statement

The original contributions presented in the study are included in the article/Supplementary material, further inquiries can be directed to the corresponding authors.

## Author contributions

WL: Data curation, Formal analysis, Methodology, Software, Writing – original draft, Writing – review & editing. G-hL: Formal analysis, Writing – review & editing, Data curation, Methodology, Software. H-hL: Data curation, Formal analysis, Software, Writing – review & editing, Investigation. P-bZ: Data curation, Formal analysis, Writing – review & editing. Y-yC: Data curation, Formal analysis, Writing – review & editing, Software. H-tS: Data curation, Formal analysis, Software, Writing – review & editing, Conceptualization, Investigation, Methodology, Project administration, Supervision. H-cC: Conceptualization, Methodology, Project administration, Supervision, Writing – review & editing, Validation.
