# Supplementary material for: Efficacy and safety of combined stent retriever and contact aspiration vs. stent retriever alone on revascularization in patients with acute ischemic stroke: a systematic review and meta-analysis
Source: Front Neurol. 2024 Jun 4;15:1365876. doi: 10.3389/fneur.2024.1365876 (PMC11183822; doi:10.3389/fneur.2024.1365876)
Supplement: Supplementary file 1 [file Data_Sheet_1.docx]

**Supplementary:**

**Search Type:**

(((((((((((((acute large vessel occlusion) OR (Ischemic Strokes)) OR (Ischaemic Stroke)) OR (Ischaemic Strokes)) OR (Cryptogenic Ischemic Stroke)) OR (Cryptogenic Stroke)) OR (Embolism Stroke, Cryptogenic)) OR (Wake-up Stroke)) OR (Wake up Stroke)) OR (Acute Ischemic Stroke)) OR (Acute Ischemic Strokes)) OR (Ischemic Stroke, Acute)) AND (((((((((((Thrombectomies) OR (Percutaneous Aspiration Thrombectomy)) OR (Aspiration Thrombectomies, Percutaneous)) OR (Aspiration Thrombectomy, Percutaneous)) OR (Percutanous Aspiration Thrombectomies)) OR (Thrombectomies, Percutaneous Aspiration)) OR (Thrombectomy, Percutaneous Aspiration)) OR (Aspiration Thrombectomy)) OR (Aspiration Thrombectomies)) OR (Thrombectomies, Aspiration)) OR (Thrombectomy, Aspiration))) AND ((Stent Retriever) OR (Stent Retriever alone))

Supplementary Table1: Inclusion of observational studies literature NOS evaluation scales

| Study | Selection | | | | Comparability  Control for important factor | Exposure | | | Scores |
| --- | --- | --- | --- | --- | --- | --- | --- | --- | --- |
|  | Adequate definition of cases | Representati-veness of the cases | Selection of controls | Definition of controls |  | Ascertainment of exposure | Same method Of ascertainment for Cases And Controls | Non-responserate |  |
| Hesse,et al 2018 [14] | 1 | 1 | 0 | 1 | 2 | 1 | 1 | 1 | 8 |
| Procházka,et al,2018[15] | 1 | 1 | 0 | 1 | 2 | 1 | 1 | 1 | 8 |
| Colby,et al,2019[16] | 1 | 1 | 0 | 1 | 2 | 1 | 1 | 1 | 8 |
| Kim,et al, 2020[17] | 1 | 1 | 0 | 1 | 2 | 1 | 1 | 1 | 8 |
| Di Maria,et al,2021[18] | 1 | 1 | 0 | 1 | 2 | 1 | 1 | 1 | 8 |
| Blasco,et al,2022[19] | 1 | 1 | 0 | 1 | 1 | 1 | 1 | 1 | 7 |
| Maïer,et al,2022[20] | 1 | 1 | 0 | 1 | 2 | 1 | 1 | 1 | 8 |
| Mohammaden,et al,2022[21] | 1 | 1 | 0 | 1 | 2 | 1 | 1 | 1 | 8 |
| Perez-Garcia,et al,2022[23] | 1 | 1 | 0 | 1 | 2 | 1 | 1 | 1 | 8 |
| Abdelrady,et al,2023[24] | 1 | 1 | 0 | 1 | 2 | 1 | 1 | 1 | 8 |
| Abecassis,et al,2023[25] | 1 | 1 | 0 | 1 | 2 | 1 | 1 | 1 | 8 |
| Bala,et al,2023[26] | 1 | 1 | 0 | 1 | 1 | 1 | 1 | 1 | 7 |
| Huo,et al,2023 [13] | 1 | 1 | 0 | 1 | 2 | 1 | 1 | 1 | 8 |
| Xu,et al,2023[27] | 1 | 1 | 0 | 1 | 2 | 1 | 1 | 1 | 8 |
